# Constipation

**Published:** 1877-08

**Authors:** 


THE condition of the human system most liable to terminate in some incurable disease, is that of habitual "constipation." And still a majority of persons not actively employed suffer from it. It should be known to everybody that "Nelaton's Suppository" will radically cure ninety-nine cases in a hundred. Where costiveness depends upon "piles," the cure is certain; no case of piles will resist this treatment for two days. Price 50 cents a box. Sent free on receipt of price. HALL & RUCKEL, 218 Greenwich Street, New York.

